# The Medical Witness for the Prosecution and the Defence
*Based on a paper delivered to the Plymouth Medical Society on 19th October, 1951, under the auspices of the British Medical Association.


**Published:** 1952-01

**Authors:** Francis E. Camps

**Affiliations:** Lecturer in Forensic Medicine, the London Hospital


### THE MEDICAL WITNESS FOR THE PROSECUTION AND THE DEFENCE*

BY

FRANCIS E. CAMPS, M.D.

*Lecturer in Forensic Medicine, the London Hospital*

While this paper was being prepared the *Lancet* published an interesting and provocative commentary on *The Expert in Court* (September 15th, 1951) reviewing the procedure in this country and in France, and suggesting that the future lies in the employment of experts as assessors rather than as witnesses. It makes, I think, a fundamental error in failing to appreciate the difference in the legal procedure of the two countries, and assumes that because things are unsatisfactory, they are incapable of improvement. It is generally appreciated that there are some unsatisfactory medical witnesses, just as there are undesirable lawyers who seek to impose their influence upon the evidence of the witness. But surely some form of disciplinary committee could deal with the former and the integrity of the witness with the latter. The disadvantage of an assessor appointed by the court is that his knowledge may become out of date, or that he may be obsessed with opinions of his own. Neither of these difficulties is likely to arise when each side has proper advice and the advisers realize that they are present to assist the court. A further difficulty is that although many people would like to advise in cases, there are not many who are capable of doing so. The solution clearly lies in a better standard of forensic medicine, for it can be just as galling to sit in court and listen to a case thrown away because of inadequate medical advice as to hear it lost by an inexperienced or incompetent counsel. On the other hand there is a real danger to medical jurisprudence in a witness who is willing to assist a case by supporting some improbable theory without at the same time considering other explanations. This happened when abdominal stab wounds which were clearly inflicted after the cessation of the circulation were explained as being caused by a man running several times upon the knife—a remarkable man to jump about with a severed abdominal aorta!

This introduction attempts to give a background to the discussion of the medical witness, whether called for the prosecution or defence, his duties, his limitations and most of all his approach to the case.

The task of the medical witness nowadays is more difficult than it was. This is mainly because the standard of forensic medicine is improving. Clearly the defence should receive as good advice as the prosecution. Also counsel is often more aware that medical evidence can be challenged. The duty of a medical witness is to place before the court the medical facts of the case, and to base

* Based on a paper delivered to the Plymouth Medical Society on 19th October, 1951, under the auspices of the British Medical Association.

an opinion on them from current knowledge and personal experience which will assist it to reach a decision. The criterion of such an opinion must be that it cannot be contradicted by a reputable colleague.

### THE PROSECUTION WITNESS

It is not on the other hand the duty of the witness to assume the role of a prosecutor or a defender. Theoretically this is easy, but it presents difficulties which are fundamental problems at the present moment, for the medical witness when called by and not for the prosecution is in a peculiar position. This is because it is frequently upon his opinion that the prosecution has been conceived, whether he be an experienced forensic pathologist in a case of murder, or a junior house surgeon in a case of common assault. Once an opinion has been expressed, either from experience or ignorance, it takes a very great man to modify it and risk the loss of prestige. Further, in the majority of cases at the time of the examination an arrest has yet to be made, or even a suspect found. Under such circumstances the examiner frequently has to make decisions and give opinions without any restraining or critical opposition, and even if these are incorrect (and the greatest can be wrong) they may still remain unchallenged in spite of the modern awareness of counsel of the weakness of many medical theories. To give a gross example, a medical witness once said that he identified semen on a handkerchief by its smell.

In Scotland two pathologists are present at the post mortem examination of cases of death from violence and they furnish independent reports which avoids a single opinion. In this country the pathologist or doctor who does the autopsy may be the only one to see the original material but he is expected to and usually does adopt an impartial attitude without indulging in speculative theories. Too much stress cannot be laid on the advantage of seeing the original material which should be preserved for further examination or photography. Recently there was a Ministry of Pensions case in which the claim depended entirely upon histological sections. The material had not been preserved at the autopsy but a dogmatic opinion had been given upon the age of a cirrhosis of the liver on naked eye appearances, which could not be disputed. It can be seen therefore that the prosecution has some initial advantage, although it must show its hand completely; the defence on the other hand can use the weapon of surprise.

Cases of assault and grievous bodily harm are quite frequent and are extremely important from the point of view of the public. In spite of this the injuries are usually reported upon by a young hospital house officer with little or no experience of the implication of his examination or opinion. By his very inexperience he is not immune to suggestion by a police officer who suffers from the common delusion that all doctors must be experts in forensic medicine. He little appreciates that most doctors have had rather less training in the subject than he himself has had at the detective training school.

The following examples show the dangers of making ill-considered statements.

1. A house surgeon at a large hospital rang up one day in a very worried state and not without reason for he had given a description to the police of some injuries he had never seen and now he had been summoned to give evidence on oath. An old lady had been brought to the casualty department with a cut head and after treatment had been admitted into his ward for the night for observation. When she was discharged the next day the dressings were still intact and at no time had he seen her injuries. Later a C.I.D. officer requested a statement of these injuries which were alleged to have been caused by an assault. He was referred to the house surgeon by the superior medical officer who had originally treated the case but had no desire to get mixed up in the affair. The house surgeon, in his ignorance, had given a signed statement based on the notes, and was summoned to attend the local magistrates court to give evidence. On re lizing that he might be cross-examined upon something he had never seen he sought advice. In the interests of the doctor, the hospital, and the police, this was unsatisfactory and it was arranged for the senior medical officer to be interviewed by a senior member of the police and a statement of what actually had been seen was obtained.

2. This is a rather more serious example, for an examination by a pathologist instructed by the defence showed that the case was far more serious than the prosecution had believed. The allegation here was that the man (the landlord of a public house) had been struck with an unbroken ginger-ale bottle resulting in a cut forehead. On examination three weeks after the injury the scar of a cut was found to extend from the hair margin on the right brow across to the outer side of the left eyebrow. The scar was firm, not indurated and very thin. Quite clearly on mechanical grounds alone, it could not have been caused by splitting by a blow from an unbroken ginger ale bottle. On the other hand it was a perfect example of an incised wound such as would be caused by a razor. As the witnesses adhered to the story of the bottle the man was acquitted.

Each contretemps could have been avoided, the first by better training in the medical schools, and the second by routine photographs of wounds, which can be submitted for advice to a more experienced colleague. Ideally, a second opinion should always be available to help the inexperienced practitioner who should not be required to shoulder such responsibility alone.

Lest it be thought that cases like this are limited to the newly qualified, the following is an illustration that even experienced doctors can find themselves in difficulties, usually due to lack of care in preparation.

A general practitioner was called to examine a man who had been found asleep in his car in the middle of a road. He unhesitatingly certified him as suffering from the effects of alcohol. At a subsequent trial at Quarter Sessions carbon monoxide poisoning from a faulty exhaust pipe was put forward as an explanation by the defence. On cross-examination the doctor agreed that the symptoms might be similar, and that he was not prepared to exclude this alternative. He neglected to realize that if the man had been overcome by the fumes and been sitting in a car for two hours with the windows shut the properties of carbon monoxide are such that he would not have been in the cell that night but in the mortuary.

Apart from the public interest the possible psychological effect on a young witness of an unfortunate encounter in the witness box must be appreciated, for no one who has experienced it will forget being " torn to pieces " and it may well colour a man's whole outlook.

When a medical witness is called to give evidence by the prosecution the examination in chief will consist of two parts, factual and opinion, and he must be prepared to support both in cross-examination with any additional information or opinion which the defence may see fit to require. The secret of success therefore lies in adequate preparation including a very thorough initial examination of whatever material is available. The autopsy must not be limited to establishing the cause of death but must include an examination of all organs with notes of both positive and negative findings. Above all it must not be modified or limited if the case appears straightforward for such are the cases which go wrong.

There was a case of murder some years ago when at autopsy the pathologist collected all the routine material such as finger-nail scrapings and hair and blood samples. At this stage no arrest had been made. He was rung up 24 hours later by the C.I.D. officer in charge telling him that a man had been charged with murder and had admitted it, and under the circumstances he proposed to throw away the material as it would no longer be needed. A firm reply was given that it must still be submitted to the laboratory, a recommendation which proved fortunate as the line of defence adopted was disproved by the laboratory findings.

At the autopsy all relevant material must be saved or photographed as a permanent record available for both prosecution and defence. The importance of this is well illustrated in the following case.

The victim of a stabbing attack fell down whilst escaping and sustained a fractured skull as well as his stab wounds. At the trial defending counsel suggested to the hospital surgeon who had been present at the autopsy that the skull was unduly thin, and he obtained some measure of agreement. In cross examination the pathologist was asked the same question and expressed as his opinion that it was within normal limits, which immediately evoked a suggestion that it was merely a matter of opinion. When however the counsel was given the opportunity of seeing the skull which was available in a box at the back of the court, he decided to accept the pathologist's evidence.

Notes on the factual evidence must be made at the time and this is best done by a trained secretary who records the findings in the mortuary. An opinion is then based on the facts and consists of a part which is non-controversial and a part which is more speculative or less capable of proof. The report gives the facts and the non-controversial opinion whilst a confidential letter to the officer in charge of the case explains the more speculative aspects which may assist him in his investigations.

There then remains only " the closing of the bolt holes " as Sir Travers Humphreys so aptly called it, meaning an examination of the evidence from the opposite side which may indicate an attempt to prove some of the more speculative opinions by experiment. Each case must always be treated as a new problem and few will be found to be wholly covered by any book on forensic medicine, many of which contain inaccuracies whilst most show gaps in their information. It will frequently be necessary to consult experts in special subjects such as biology or anatomy and this should be done without hesitation.

In the case of Stanley Setty several of such problems occurred and a most interesting example was the question of the blood under the floor-boards in the dining room of Hume's flat. This raised the question as to how much this blood beneath the boards would represent in spillage on top. It was estimated that the amount of blood beneath the boards was a teacupful and it was decided to try a simple experiment which had to be planned so as to give the maximum of benefit to the defence. As fluid blood was necessary it was collected from a fatal case of carbon monoxide poisoning and a pint poured in two places on a floor selected for its similarity to the original. One pint was allowed to rest for 10 minutes and w s then mopped up, the other was allowed to remain overnight. On taking up the boards quantities were obtained comparable to those in the original house. Not only was a rough estimate of the volume obtained from this, but it was learnt also that if blood is left overnight it required an abrasive as well as water to remove the stain from the floor while if it is mopped up at once removal is easy. No dilution was found of the blood beneath the floor which indicated that it had been cleaned up reasonably soon after being spilt.

With proper preparation it should be possible to give evidence fairly, together with a non-controversial opinion. It does not follow that it will necessarily help the prosecution, as occurred in *Rex v. Boyle.* Here the pathologist was asked whether he would expect the assailant who had inflicted incised wounds on the neck and hands of the girl to be contaminated with blood. In view of the extent of the wounds and surrounding blood at the scene of the attack it was obviously a probability. The absence of blood on the accused when arrested some time later and the fact that nobody had noticed blood on his clothes was made a strong point by the defence and may have influenced the result, an acquittal.

In a slightly different manner in the case of Setty, the defence made great play upon the absence of defence wounds on the hands as an indication that more than one person was involved. This is obviously one possible explanation and was stressed in evidence by the pathologist called by the defence. Many alternative explanations existed; he might have been stunned first, or been drunk, or had his hands in his pockets or caught with his coat over his shoulders (a well-known trick). Such an opinion is allowable for the defence but not for the prosecution.

On the other hand it is unjustifiable for an expert witness to postulate a medical theory of his own unbacked by generally accepted medical knowledge, and by using his prestige to discredit other evidence; or putting it more bluntly to make statements in a court of law which would not be accepted at a medical meeting. Such evidence was called in a murder trial in which the dead man after being attacked with a hammer and sustaining multiple fractures of the skull had recovered after trephining except for some slight paresis. He died suddenly whilst still confined to bed 10 days later from a pulmonary embolus from a phlebothrombosis of his right popliteal vein. This, said the defence medical expert, was due to a disease of the vein (not specified) and had nothing to do with confinement to bed or the head injury. He based this opinion upon a long experience of head injuries in which he had never met either a phlebothrombosis or pulmonary embolus following one. This medical dogma was not confirmed by the records of one neurosurgical unit in which cases of pulmonary embolus had occurred following head injuries.

To summarize, the duty of the medical witness for the prosecution is to be thorough in examination, critical in evaluation and careful in opinion. Above all he must not take things for granted and must check up on both theory and fact. He should shut the bolt holes but not press his evidence.

### THE DEFENCE WITNESS

The medical witness for the defence is placed in a more difficult position because however carefully he tries he must always be in danger of being prejudiced. His greatest value is, of course, to study the prosecution witness, and his opportunity arises when that witness has by lack of care left a large gap, or by over-confidence overstated an opinion. If the expert witness for the prosecution has done his duty the expert for the defence can resort only to quibbles, a practice which is responsible for damaging the reputation of medicine in the eyes of members of the legal profession. It is far better to say that there is no defence. Frequently counsel have themselves to blame by failing to appreciate that medicine is not an exact subject, for they would like to believe that human beings in their make-up and disease conform to the specification of textbooks, in the same way as mass-produced cars do to blue-prints. This fact must be stressed when advising on the medical aspects of any case. An element of surprise in presenting expert evidence for the defence may undoubtedly help the accused, but how far it is ethically justifiable to make use of this fact is a matter of opinion. It is, of course, fascinating to be asked to advise the defence in a criminal case but in order to do it properly it is essential to have all the factual evidence available. Depositions, couched in lay language, are sometimes not helpful. For example, evidence stating that the findings are in keeping with death from asphyxia may be satisfactory to the layman who has not the slightest desire to hear about such things as Tardieu's spots and the like, but the medical adviser must know more particulars of these " findings ", because to his experienced eye they might well not seem to be in keeping at all.

At a meeting sometime ago one of my colleagues said that he could not obtain the factual information he desired when advising the defence and felt seriously handicapped. This should not be so and the procedure to follow is quite simple. The instructing solicitors should be asked to approach the Director of Public Prosecutions for permission for the pathologist to see any documents and if necessary speak to the pathologist who is appearing for the prosecution. Such permission is never withheld and it is thus possible to obtain an exact and detailed record of the post-mortem findings. On the other hand a copy of a post-mortem report has been refused by a coroner on the grounds that as it had not been produced in evidence (the cause of death only was taken at the inquest) it was not a proved document. This seemed strangely illogical in that a pathologist can be present at a post-mortem examination on behalf of a man who has already been charged, but cannot obtain access to the report if the man has the misfortune to be arrested after the autopsy has been performed. The explanation, however, proved to be quite simple for it appeared that the pathologist had expressed opinions in the report which he did not wish to reveal prematurely in case he should desire to modify them later when further facts became available. Since it is only the factual evidence that is required by a defence witness the difficulty could easily be solved by restricting the report to non-controversial opinions which will not require amending.

When all the information has been obtained, the medical aspects of the case for the prosecution are examined for possible flaws of any importance, and an opinion submitted. Once again I must stress that if there are no flaws this should be stated without prevarication.

A case was submitted for opinion in which a man had been charged with driving under the influence of alcohol. His car had had a slight impact with a passing lorry in a narrow street and he had been found to be unsteady on his feet with his breath smelling of alcohol. He was taken to the police station and seen by a doctor who took fifty-seven minutes to examine him and then certified him as unfit to drive a car. A sample of blood submitted to the laboratory was found to contain about 0·25 per cent. of alcohol and a parallel figure was found in the urine. At the Magistrates' Court he had elected to go for trial to Quarter Sessions. His solicitors had put a footnote against the length of time taken over the medical examination " ? uncertain ". The explanation was that far from being uncertain the doctor was clearly making quite certain by the thoroughness of his examination. The intake of alcohol as shown by blood and urine was so high and the technique of collection so unassailable that there was no defence from the medical point of view. It was a great temptation to make the suggestion that with a level such as he had he was incapable even of giving consent to the examination!

It is most undesirable for medical witnesses to use some pet theory to confuse the court, a practice which can only lead our profession into disrepute. Sir Travers Humphreys felt so strongly about this after the Haigh case that when approached for permission in a poor persons case to obtain expert medical advice he stipulated that the medical experts should consult one another and attempt to agree an opinion so as to avoid the unpleasantness and waste of time caused by a conflict of medical opinion on trivial points.

During the summer I had the opportunity of reading a book called " Court Room " by Quentin Reynolds, which is the story of Leibowitz, a counterpart of Marshall Hall at the American Bar, who defended the Scottsboro' boys and later became a judge. What particularly fascinated me was the fact that his success was based upon three essential points.

1. Always putting himself in the position of the opposition.
2. Never leaving any point unexamined.
3. The fundamental principle that it is practical experience that counts.

And this summarizes the preparation and presentation of any case by the medical witness if it is to be done properly. I would add that if medical witnesses would all maintain the same standard as is expected at a medical meeting there might be fewer references to the necessity for a thick skin, and fewer criticisms of the legal profession.

Reverting to my opening remarks, I personally do not think that a panel of expert witnesses, one of whom can be nominated by a judge, would necessarily fulfil the requirements of our courts. I do however think that a panel of approved pathologists from whom a selection can be made by solicitors would be a definite advance. At the same time a minimum fee should be established and the defence solicitors not left to rely upon personal friendship, or mutual sacrifice, or the generosity of the taxing master in poor persons cases. The defence should be entitled to as high a standard of opinion as is the prosecution. Above all I do believe that the reputation of medical witnesses can only be maintained by high standards of self-criticism.

